# Application of Nanomaterials in Endodontics

**DOI:** 10.34133/bmef.0043

**Published:** 2024-04-17

**Authors:** Farzaneh Afkhami, Yuan Chen, Laurence J. Walsh, Ove A. Peters, Chun Xu

**Affiliations:** ^1^ School of Dentistry, The University of Queensland, Brisbane,QLD4006, Australia.; ^2^ Sydney Dental School, Faculty of Medicine and Health, The University of Sydney, Camperdown, NSW 2006, Australia.; ^3^ School & Hospital of Stomatology, Wenzhou Medical University, Wenzhou, Zhejiang 325027, China.; ^4^ Charles Perkins Centre, The University of Sydney, Camperdown, NSW 2006, Australia.

## Abstract

Recent advancements in nanotechnology have introduced a myriad of potential applications in dentistry, with nanomaterials playing an increasing role in endodontics. These nanomaterials exhibit distinctive mechanical and chemical properties, rendering them suitable for various dental applications in endodontics, including obturating materials, sealers, retro-filling agents, and root-repair materials. Certain nanomaterials demonstrate versatile functionalities in endodontics, such as antimicrobial properties that bolster the eradication of bacteria within root canals during endodontic procedures. Moreover, they offer promise in drug delivery, facilitating targeted and controlled release of therapeutic agents to enhance tissue regeneration and repair, which can be used for endodontic tissue repair or regeneration. This review outlines the diverse applications of nanomaterials in endodontics, encompassing endodontic medicaments, irrigants, obturating materials, sealers, retro-filling agents, root-repair materials, as well as pulpal repair and regeneration. The integration of nanomaterials into endodontics stands poised to revolutionize treatment methodologies, presenting substantial potential advancements in the field. Our review aims to provide guidance for the effective translation of nanotechnologies into endodontic practice, serving as an invaluable resource for researchers, clinicians, and professionals in the fields of materials science and dentistry.

## Introduction

Nanotechnology has experienced notable advancements, capturing the attention of various scientific disciplines including dentistry [1]. In endodontics, the application of nanomaterials is reflected in the growing body of research literature [2–4]. As for the definition of nanomaterials, the European Commission recommends an updated definition, proposing that nanomaterials encompass solid particles of natural, incidental, or manufactured origin and at least 50% of their number-based size distribution falls within the range of 1 to 100 nm [5]. Alternatively, nanomaterials may exhibit an elongated shape, with two external dimensions smaller than 1 nm and one dimension larger than 100 nm. They can also adopt a plate-like configuration, with one external dimension smaller than 1 nm and the remaining dimensions larger than 100 nm [5]. For the United States Food and Drug Administration (FDA), nanomaterials are defined as materials or products at the atomic, molecular, or macromolecular level, with at least one dimension influencing their functional behavior within a length scale of approximately 1 to 100 nm. The implementation of a functional nano-system encompasses the design, fabrication, and deployment of structures and devices that exhibit novel properties and functions due to their small size, along with the control or manipulation of the product at the atomic level [6].

The properties of materials, including shape, texture, and size, exert a profound influence on the biological functions. At the nanoscale, materials exhibit distinct and innovative properties that are not observed in their atomic or macroscopic bulk states. This phenomenon, known as the “size effect,” arises from the nanoscale dimensions of nanomaterials. Such dimensions contribute to a large proportion of surface atoms, elevated surface energy, spatial confinement, and a decrease in structural imperfections, forming characteristics that diverge markedly from those found in bulk materials [7]. For example, nanoparticles (NPs), a type of nanomaterial characterized by a spherical morphology and nanometer-scale size, exert antibacterial effects through mechanisms distinct from traditional antimicrobial therapies [8,9]. NPs with positive charges and larger specific surface areas effectively interact with negatively charged bacteria, leading to heightened antibacterial activity compared to bulk materials [10,11].

**Fig. 1.**
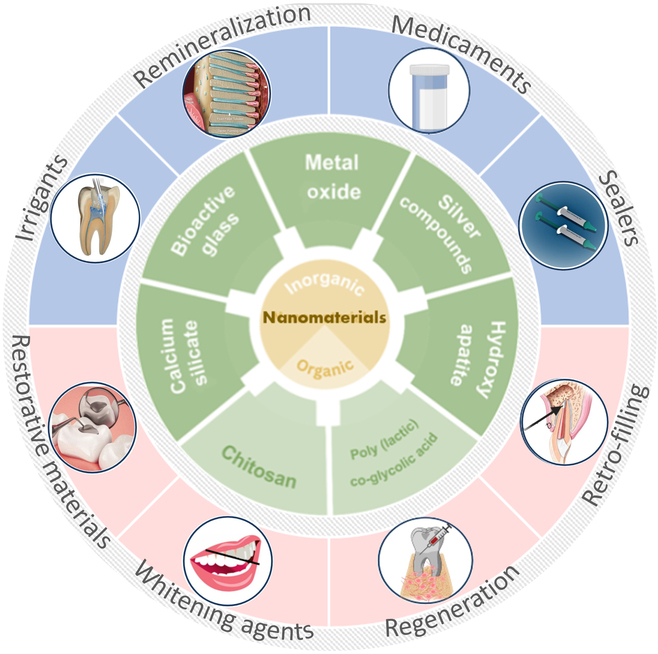
Utilization of nanomaterials in endodontic applications.

Owing to their superior properties and advantages over conventional materials, nanomaterials have gained remarkable traction in endodontics (Fig. 1). Recent endeavors have explored the application of NPs capable of releasing reactive oxygen species (ROS) for the remediation of discolored teeth, a methodology elaborated by Kim *et al.* [12]. Prolonged and intensive research has been dedicated to amalgamating endodontics with nanotechnology, with a specific focus on integrating nanoscale materials into restorative resin composites and mineralization agents, as detailed in [13–18]. Moreover, nanomaterials offer substantial enhancements to root canal procedures by augmenting the efficacy of antimicrobial strategies and by improving the quality of obturation materials [19].

Additionally, contemporary studies have proposed nanomaterials as a viable alternative to antibiotics, aiming to mitigate the burgeoning issue of antibiotic resistance [20]. In the realm of tissue engineering, the strategic integration of nanomaterials and nanofeatures into scaffolds promises to revolutionize regenerative approaches, enabling the comprehensive restoration of the dentin–pulp complex in therapeutic settings [19]. In conclusion, nanotechnology’s integration into endodontics signifies a leap toward refining techniques, materials, and the endodontic armamentariums, with the ultimate objective of achieving superior treatment outcomes [21].

This review article provides a comprehensive overview of the diverse applications of nanomaterials in endodontics. We systematically outline their utilization across various domains including medicaments, irrigation solutions, obturation, sealing, retro-filling, root repair, and pulpal regeneration. We underscore their capacity to augment antimicrobial efficacy, enhance mechanical attributes of endodontic materials, and foster tissue regeneration. Furthermore, we offer insights into prospective research trajectories within this domain, pinpointing areas ripe for exploration and advancement. Our endeavor is to furnish a roadmap for materials scientists, engineers, biologists, dentists, and endodontists, facilitating the seamless integration of nanomaterials into the spectrum of endodontic.

## Nanomaterials Used in Endodontic Medicaments

The objective of current endodontic treatment includes the thorough removal of bacteria, their byproducts, and pulpal remnants from infected root canals. Endodontic medicaments are considered a crucial step in effectively eradicating bacteria within the root canals. The most commonly used medication in endodontics is calcium hydroxide [Ca(OH)₂], which is effective due to its alkalinity [22–24]. The released hydroxyl ions cause cell membrane distortion, protein denaturation, and DNA damage [25]. However, the effectiveness of Ca(OH)₂ depends on the formulation, which in turn affects the pH value and the extent of penetration through dentine tubules [24]. Additionally, water-based Ca(OH)_2_ medicaments may not be able to effectively eliminate bacteria within biofilms. This was shown in a recent study for biofilms of *Enterococcus faecalis* [25].

Another challenge in achieving effective endodontic disinfection is to deal with biofilms that are located in regions that are difficult to access. During instrumentation of the root canal, some root canal surfaces remain untouched. Bacteria may persist in apical portions of prepared root canals even after irrigation procedures [26]. Additionally, microbial leakage can occur over time since the root canal has been filled [27]. Several nanomaterials, including organic NPs (mainly chitosan NPs) and inorganic NPs such as metal/metal oxide NPs and calcium-silicate NPs, have been utilized as endodontic medicaments to enhance the ability to eliminate endodontic biofilms. These are summarized below.

### Chitosan NPs

Chitosan is a cationic polymer derived from chitin, and is one of the most abundant biopolymers in nature [28]. Chitosan is biocompatible and biodegradable. It can exert antimicrobial actions and can bind to calcium within dentine [29,30]. The antimicrobial actions vary according to the molecular weight and pH, with lower pH values enhancing this activity [29–31]. Positively charged chitosan can bind to negatively charged bacteria, causing membrane damage, increased membrane permeability, osmotic leakage, and ultimately bacterial cell death [29]. In addition, chitosan can prevent bacteria from adhering to dentine [32].

Incorporating chitosan NPs into Ca(OH)_2_ products can enhance their antimicrobial activity and reduce the likelihood of recolonization of dentine with bacteria following endodontic treatment [33]. Chitosan can also be used in a standalone medicament without Ca(OH)_2_ [34], but may be most useful when used with it. A study that investigated combining Ca(OH)_2_ with chitosan NPs, chlorhexidine gluconate (CHX), or glycerine, for use as an intracanal medicament, showed the highest antibacterial efficacy against *E. faecalis* for chitosan NPs. This combination also had the highest pH and released the most calcium ions over 14 days [35]. Similar results were observed in another study that assessed calcium ion release and changes in pH, where chitosan NPs helped maintain an alkaline pH and facilitated sustained calcium ion release within the root canal [36]. Chitosan NPs can exert antimicrobial actions against *E. faecalis* biofilms, as shown in studies using these NPs at concentrations of 250 μg/ml [37]. Not only capable of inhibiting the growth of *E. faecalis* and *Streptococcus mutans,* chitosan NPs are also able to exert fungicidal activity against *Candida albicans* [34]. Moreover, chitosan may support or reinforce dentinal collagen by binding to it through its many amino and free hydroxyl groups, and this may enhance root fracture resistance [38]. However, the penetration of chitosan into dentine can be limited due to its tendency to agglomerate, even in nanosize form.

### Metal/metal oxide NPs

Silver NPs (Ag NPs) can cause damage to bacterial cells walls, and also penetrate to reach the cytoplasm, where they interact with biomolecules containing sulfur and phosphorus, such as nucleic acids [4]. In addition, Ag NPs release Ag ions that further disrupt bacterial metabolic activity [4]. Ag NPs can be delivered in various vehicles, including hydroxyethyl cellulose polymer, polyethylene glycol, and carbomer polymer. When assessed for antibacterial effects against *S. mutans, E. faecalis*, *Escherichia coli*, *Pseudomonas aeruginosa*, *and Staphylococcus aureus,* the hydroxyethyl cellulose polymer gel gave the best handling characteristics and had superior performance as an antibacterial agent [4]. The surface charge (zeta potential) of Ag NPs affects their antibacterial activity and their interactions with human cells. In one study, Ag NPs with a positive charge displayed antimicrobial activity against *E. faecalis* and good levels of biocompatibility with L929 fibroblast cell lines [39]. Other studies have demonstrated that Ag NPs give sustained release of Ag^+^ ions, which drive persisting antimicrobial effect against *E. faecalis* biofilms over 9 days [40].

As well as being used alone, Ag NPs can be added into other intracanal medicaments to enhance their effectiveness. A useful combination is Ag NPs with Ca(OH)_2_, which can significantly impair the growth of *E. faecalis* biofilms [26]. This combination also shows enhanced anti-inflammatory actions [41] and antioxidant activity [41], as well as antibiofilm actions, even when used for short-term applications [42]. It is essential that, when Ag NPs are combined with other materials, the carrier is able to release the Ag NPs. This problem has been seen with methyl cellulose gels, where release of Ag NPs is minimal [43].

Little is known about the mechanisms of adaptation to Ag NPs by endodontic bacteria, which may include intrinsic (efflux pumps, down-regulation of porins, chromosomal resistance genes) or extrinsic (point and adaptive mutations, plasmids with resistance genes) systems [44]. A recent report of silver resistance genes in endodontic pathogens raises the concern for endodontic use of Ag NPs. Bacteria present in secondary endodontic infections frequently have plasmids that carry genes for antibiotic resistance, and these same plasmids are now also recognized to also carry genes for resistance to certain metals such as silver. Due to the presence of such resistance genes, the use of metal NPs (especially Ag NPs) in endodontic treatment needs careful assessment in the context of resistance [45].

Similar to silver, copper NPs (Cu NPs) also exert antimicrobial actions against Gram-positive and Gram-negative bacteria as well as fungi [46]*.* They penetrate bacterial cell walls and then bind to sulfhydryl (-SH groups), causing protein denaturation and enzyme inactivation. Cu NPs also indirectly impair the synthesis of DNA and proteins in bacteria [47]. Besides an immediate action, Cu NPs also exert a longer-term antimicrobial action over 7 days. This makes them of interest against multi-species biofilms [47]. Cu NPs cause greater generation of ROS than ZnO NPs, making them superior against many strains of bacteria [48]. The released ROS penetrate through biofilms, causing inactivation of bacteria even in the deeper layers [49].

In addition to metal, zinc oxide NPs (ZnO NPs) exert antimicrobial actions against a wide range of microorganisms through the generation of ROS and penetration of NPs through the outer cell membrane or into the cytoplasm. ZnO NPs can inhibit the growth of *C. albicans* [50] as well as *Porphyromonas gingivalis*, *Prevotella intermedia*, *Fusobacterium nucleatum*, *Aggregatibacter actinomycetemcomitans* [51], and *E. faecalis* [52]. ZnO NPs are superior to microparticles of ZnO when added to Ca(OH)_2_, giving enhanced release of hydroxyl ions and improved antibacterial activity against *E. faecalis* [53]. The addition of ZnO NPs to Ca(OH)_2_ makes such combination medicaments of interest as intracanal medicaments [54]; however, adding ZnO NPs may not be as useful as adding other materials, such as chlorhexidine [55].

### Calcium-silicate NPs

Calcium-silicate NPs such as Ca(OH)₂ NPs, bioactive glass, and mesoporous silica NPs are also used for endodontic medicaments. Compared with conventional micrometer-sized particles, Ca(OH)_2_ NPs (<100 nm) are considered to penetrate deeper into dentinal tubules, and this gives improved antimicrobial actions against *E. faecalis* at depths up to 400 μm within dentinal tubules [56]. These NPs when in the size range of 80 to 115 nm did not cause any change in fracture resistance of the teeth over 1 month [38]. NPs of Ca(OH)_2_ are more effective at eliminating *E. faecalis* than NPs of calcium oxide, when tested in blocks of dentine [57]. Ca(OH)_2_ NPs appear to be a safe and effective alternative to conventional Ca(OH)_2_ and cause no significant adverse effects on dentine microhardness [58], but will cause subtle changes in the chemical structure of superficial layers of dentine, as evidenced by an increase in the phosphate/amide I ratio [58].

Bioactive glass contains SiO_2_, CaO, Na_2_O, and P_2_O_5_ and is reported to have antimicrobial actions. Antimicrobial effects of bioglass NPs on *E. faecalis* biofilms are superior to those of larger particles of bioglass [59]. Mesoporous bioactive glass (MBG) is a new class of bioglass with a well-ordered mesoporous channel structure and pores that can be loaded with various materials to enhance the biological activity of the NPs [60,61]. Silver-loaded MBG NPs give sustained release of Ag ions, making them effective against planktonic bacteria [62] as well as biofilms [62]. In addition to MBG NPs, mesoporous silica NPs were also reported to be loaded with CHX, and under alkaline conditions, they can release CHX, as well as calcium and silicate ions, in a sustained manner. Such NPs can exhibit strong antimicrobial actions against *E. faecalis*. They can also promote mineral formation [63]. In a similar manner, mesoporous calcium-silicate NPs can also be loaded with silver to give sustained release of Ag^+^, Ca^2+^, and SiO_3_^2−^ ions. The released silver ions can then interfere with the growth of endodontic bacteria, such as *E. faecalis* [64].

## Nanomaterials Used as Endodontic Irrigants

Irrigation is a key aspect of endodontic disinfection as it can reach, inactivate, and dislodge bacteria attached to dentine walls that have not been reached by instruments. Thus, irrigation facilitates both the killing and removal of microbes, as well as the removal of remnants of necrotic tissues and dentine debris [65]. To date, common choices for irrigants with antimicrobial activity for use in root canal treatment are sodium hypochlorite (NaOCl), CHX, and hydrogen peroxide [66]. CHX is less irritant than NaOCl should accidental extrusion occur; however, it cannot dissolve organic matter. Its lack of proteolytic action necessitates the use of NaOCl as the main irrigant.

In an ideal scenario, a nontoxic or low-toxic irrigant with sufficient antimicrobial ability would address concerns about adverse reactions caused by inadvertent extrusion. NPs have been explored as possible alternatives, especially Ag NPs [4,8,67]. NPs have a high surface area-to-volume ratio that increases the interactions with microbes, and can be designed to have particular sizes and shapes that enhance their antimicrobial actions [68]. It was reported that Ag NPs have high biocompatibility for human tissues, but are highly toxic for bacteria, including *E. faecalis* [69,70]. Moreover, as shown by optical coherence tomography, Ag NPs can reach the apical third of the root canal when used in endodontic irrigants [71]. Ag NPs (1 to 2 nm in size) were found to penetrate the smear layer and form a barrier film on the dentinal surface, which would potentially lower the risk of later bacterial invasion [72], without harming the physical structure of the dentine itself [73,74].

An important challenge is the delivery of irrigants into regions of the root canal system with complex anatomy [75]. Chitosan will form deposits on dentine surfaces [76]. To drive penetration further into the root canal, low-level electric fields have been used to facilitate the movement of cationic chitosan NPs. This approach causes chitosan NPs to form a thin homogeneous layer in the apical 2 mm of the root canal system, with an associated reduction in bacterial load [77]. This concept has been extended to include actively propelled NPs to target locations that are hard to reach. One example includes using oscillating magnetic fields to propel helical nanobots with an iron-cored silica blob-head to travel through dentinal tubules, with magnetic hyperthermia being used to kill bacteria [78].

Moreover, NPs can be used in conjunction with various activation methods to enhance bactericidal effects against *E. faecalis* and other prominent pathogens featured with high resistance to disinfectants. It has been reported that activation methods including manual dynamic activation (MDA), passive ultrasonic irrigation (PUI), and sonic irrigation can improve irrigant penetration across different regions of the root canal [79]. Ag NPs-graphene oxide irrigants under ultrasonic activation exhibited improved efficacy against multispecies biofilm compared to syringe-mediated irrigation and XP Endo Finisher file-mediated irrigation [80]. Another promising way to optimize irrigation is to introduce laser-assisted activation. Near-infrared (NIR) laser energy itself can have disinfecting actions deep in dentine and impedes the re-colonization of biofilm due to its capability to occlude dentinal tubules [81]. Nanocurcumin photosensitizers and light-emitting diode (LED) were combined as a means of photodynamic therapies (PDTs) to eliminate *E. faecalis* from the root canal system. Along with the shock wave enhanced emission photoacoustic streaming technique, this combination therapy had improved efficacy against *E. faecalis* [82]. Diode lasers (DLs) have been combined with chitosan NPs to achieve bacterial eradication [83]. Ag NPs, together with Nd:YAG lasers, showed greatest reduction of *E. faecalis,* giving superior effects to using Ag NPs alone [84]. Another study compared the effectiveness of Ag NPs, an 810-nm DL, conventional PDT with indocyanine green (ICG) photosensitizer, modified PDT with Ag NPs in disinfecting root canals infected with *E. faecalis*, and found significant reductions in bacterial colony counts across all groups, with the greatest reduction observed in the Ag NPs/ICG/810-nm DL group, suggesting the potential use of PDT with Ag NPs, DL, and Ag NPs as an adjunct for root canal disinfection [85]. Although diverse lasers are available, the wide range of laser parameters reported in various studies leads to a dilemma where there is no universally agreed standardized disinfecting protocol at the present time [86]. One recent study further compared the efficacy of different systems to activate Ag NP irrigant for eliminating *E. faecalis*, and results showed that photon-induced photoacoustic streaming and passive ultrasonic irrigation led to higher antibacterial efficacy when compared with DL-activated irrigation and MDA [87]. Further investigations are necessary to identify whether and what kind of activations along with nanomaterials have an indispensable role in elimination of microorganisms from the root canal system.

NPs may also be of use for smear layer removal. Chitosan is a metal chelator, even at concentrations as low as 0.2% by weight [88]. Using chitosan may enhance the penetration of root canal sealers into dentinal tubules [89]. The smear layer removal capabilities of chitosan NPs are now attracting considerable interest [32]. In one study, chitosan NPs (0.2%) were as effective for eliminating smear layer as 17% EDTA, but caused less erosion, leaving the canal walls with greater microhardness and lower surface roughness [90]. In another study, chitosan NPs (0.5%) were superior to both 17% EDTA and 10% citric acid for removal of smear layer in the apical third [91]. However, this capability of smear layer elimination was inhibited at the apical third when chitosan NPs were at a higher concentration (2%).

In addition, NP-based irrigants can potentially improve the fracture resistance of endodontically treated teeth [92]. Chitosan NPs inhibit the enzymatic degradation of collagen, which can thus help to conserve the physical properties of dentine [93], and stabilize the structural integrity of the root over the longer term [94,95]. This adds to the previously mentioned benefits of using chitosan NPs in irrigation fluids to lower bacterial loads and remove smear layer.

## Nanomaterials Used as Obturating Materials

**Fig. 2.**
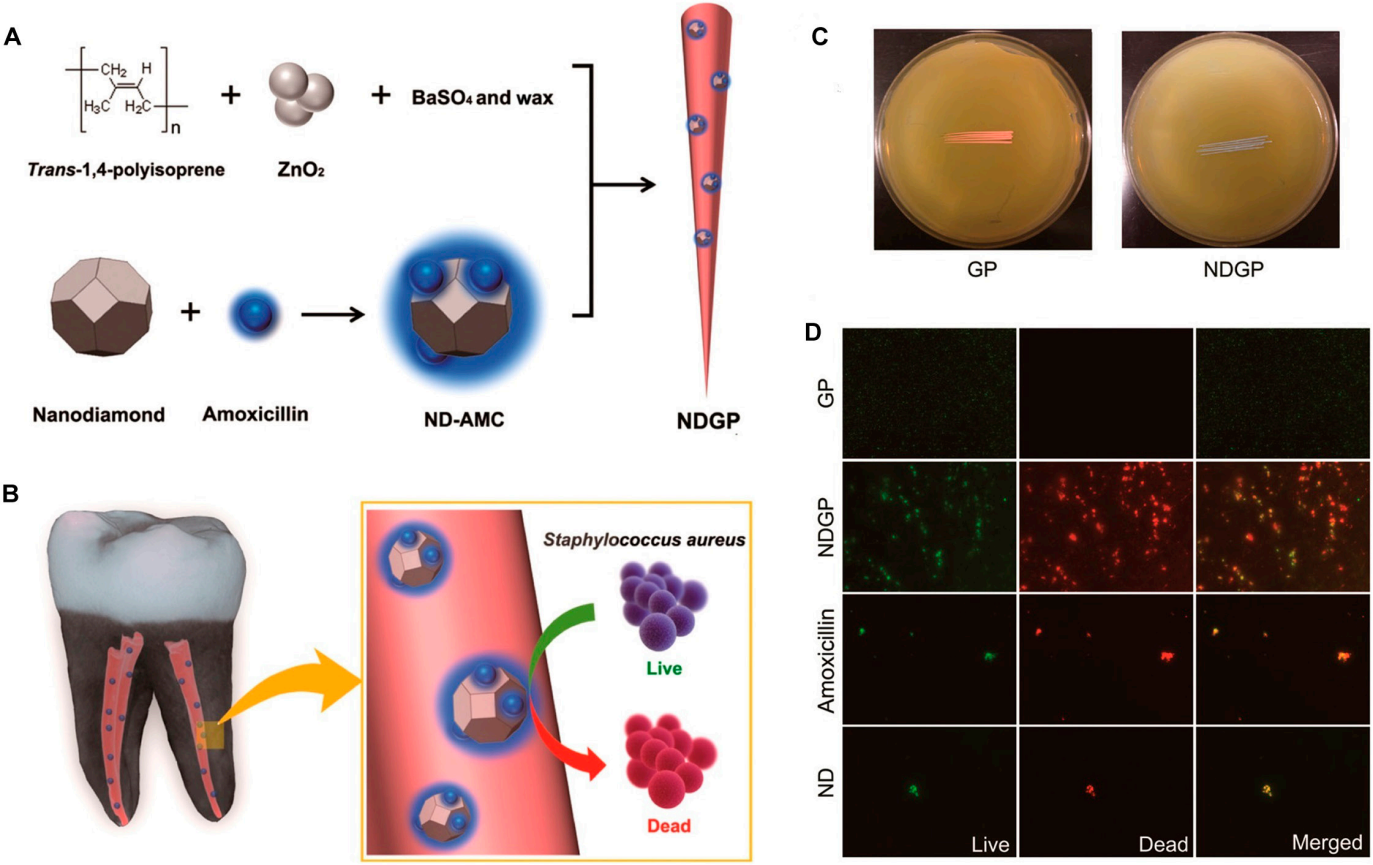
Nanodiamond incorporated gutta-percha for root canal filling. (A and B) Schematic drawing showing the synthesis and structure of nanodiamond incorporated gutta-percha, amoxicil. (C and D) Antibacterial properties of nanodiamond-incorporated gutta-percha. The image is reproduced from Lee *et al.* [99] with the permission of the American Chemical Society.

Obturating materials are important in endodontics to fill the root canal. Gutta-percha is the most commonly used root filling material [96]. It contains both organic and inorganic components. The organic components, such as natural latex isoprene polymers and waxes, provide flexibility and compressibility to the material. The inorganic components, such as zinc oxide, provide radiopacity and serve as fillers; however, they also make the material more brittle [97]. Despite its widespread use, gutta-percha has several shortcomings, notably the absence of antibacterial properties. Consequently, there is a growing interest in incorporating NPs into gutta-percha points to address these limitations [98]. Lee *et al.* [99] developed gutta-percha incorporating nanodiamond–amoxicillin conjugates, where the nanodiamond particles (4 to 6 nm) exhibit inherent antibacterial properties and adsorb antibiotics, facilitating the elimination of persistent bacteria. This nanodiamond–gutta-percha composition can lower the risk of re-infection and concurrently enhance the mechanical strength of gutta-percha points, thereby improving their clinical manageability (Fig. 2).

Ag NPs can be incorporated into gutta-percha to achieve sustained release of silver ions, imparting antibacterial properties. Diverse techniques have been employed for coating Ag NPs onto gutta-percha points, with one approach involving the use of a plasticizer [100]. The Ag NPs-coated points have low toxicity to human cells and good biocompatibility [101]. The coating exerts a worthwhile antibacterial effect against *C. albicans* as well as against several bacteria, including *E. faecalis*, *S. aureus*, and *E. coli* [102]. The treated points inhibited the growth of *E. faecalis* in a dose-related manner. An alternative approach used polyethylene glycol and polyvinylpyrrolidone as adhesives to bind silver/curcumin NPs to gutta-percha [103]. This gave antibacterial activity against *E. faecalis*, *E. coli*, and *S. aureus*. Issues with coating points are that binders may cause allergic reactions or inflammation, while water-soluble polymers may dissolve in body fluids. In addition, most Ag NPs will be embedded in the binder matrix rather than on the surface, which limits their activity [104,105]. To overcome these issues, an in situ method has been used to establish a layer of Ag NPs on the surface to give strong adhesion of the Ag NPs without the need for a binder. A higher loading of particles on the surface gave greater effectiveness against *E. faecalis* and *E. coli* [3].

Bioceramic calcium silicate NPs can be coated onto or incorporated into gutta-percha points to improve the quality of obturation. These points are used with hydrophilic bioceramic sealers, which react with moisture. The setting reaction of the sealer causes a slight expansion, which enhances the seal [98]. In one study, gutta-percha points were modified by incorporating bioceramic calcium silicate NPs both inside, and by also coating their surface. Using these modified points with BS or with an epoxy resin sealer (AH Plus) to fill root canals increased the resistance of roots to fracture of the prepared roots when compared to roots that were prepared but not filled to reach the level of intact sound roots. The treated points gave better obturation, and there was improved sealer penetration, as well as enhanced push-out bond strength [106].

## Nanomaterials as Endodontic Sealers

Root canal sealers are an integral part of the obturation process since they fill the space between bulk fillers (such as gutta-percha points) and dentinal walls, and should flow into and occlude lateral and accessory canals, thus ensuring a complete seal of the root canal system in three dimensions [107]. To ensure the long-term success of root canal treatment, endodontic sealers should provide a hermetic seal and have a stable volume over time, exert bacteriostatic or bactericidal properties while remaining biocompatible to periapical tissues, and remain insoluble in tissue fluids after setting yet be capable of being removed when necessary, in line with the specifications of ISO 6876 and ANSI/ADA 57.

Over recent decades, many sealers have been developed. Based on their prime constituent and/or reaction system, endodontic sealers can be categorized as zinc oxide-eugenol, Ca(OH)₂, calcium phosphate, glass ionomer, calcium silicate, salicylate, methacrylate resin, silicone, epoxy resin, and others [108]. No existing sealers fulfill all the requirements stated by Komabayashi *et al.* [109]. Hence, there is interest in incorporating nanomaterials to overcome these deficits. Nanomaterials themselves might possess innate functionalities to enhance the performance of certain sealers. In addition to providing sustained antimicrobial actions, they could act as carriers for the therapeutic payloads and surface modifiers [110]. NPs could also enhance the sealing ability and the process of micromechanical adhesion to dentine. They could also enhance the bioactivity in terms of mineralization. A summary of past work involving adding nanomaterials to sealers is presented in Table 1.

**Table 1.** Recent advances in nanomaterials used in endodontic sealers

Type	Base materials	Nanomaterials	Properties assessed	Main findings	Year	References
Zinc oxide-eugenol (ZOE)	Endomethasone N (Septodont, France)	AgVO_3_ nanomaterial	Biocompatibility (human gingival fibroblasts)	A reduction in cell viability due to AgVO_3_.	2021	[229,230]
	Lab-made experimental materials*	Nano-ZOE	Biocompatibility (L929 cells), sealing ability, and post-treatment pain control	The biocompatibility of the nano-ZOE was comparable to Pulpdent but lower than AH-26. In addition, nano-ZOE showed less leakage than AH-26 and commercial ZOE. However, almost half of the subjects treated with nano-ZOE reported severe to very severe pain in the first 6 h of follow-up.	2015, 2013, 2020	[231–233]
	Lab-made experimental materials	ZnO NPs	Physiochemical properties	Addition of 25% ZnO NPs significantly improved dimensional stability.	2016	[130]
Calcium hydroxide	Apexit Plus (Ivoclar Vivadent, Liechtenstein)	Chitosan NPs and ZnO NPs	Antimicrobial activity (*E. faecalis*)	ZnO NPs had superior antibacterial activity to chitosan NPs.	2018	[159]
Calcium silicate	Lab-made experimental materials (β-dicalcium silicate)	Attapulgite (ATT) nanofibers	Physiochemical properties	ATT (1–4%) enhanced the compressive strength and retarded bio-dissolution.	2022	[152]
	BioRoot RCS (Septodont, France)	Multi-walled carbon nanotubes, titanium carbide nanopowder, and boron nitride nanotubes	Physiochemical properties	The incorporation of nanomaterials enhanced compressive strength and shortened the setting time.	2021, 2020	[153,234]
	EndoSequence BC Sealer (Brasseler, USA)	Chitosan-hydroxyapatite nanocomplexes	Physiochemical properties	Addition of nanocomplexes increased nanohardness and elastic modulus.	2022	[163]
	Bright Endo MTA Sealer (Genoss, Korea)	Bioactive glass NPs	Bioactivity	Osteogenic differentiation was stimulated by the addition of bioactive glass NPs (0.5% and 1%).	2022	[140]
	Lab-made experimental materials*	Silver/zinc-loaded mesoporous Ca–Si nanoparticles (MCSNs)	Antimicrobial activity (*E. faecalis* and animal root canal infection model) and biocompatibility (MC3T3-E1 cells)	Ag/Zn ratios influenced antibacterial activity and cytotoxicity. Ag/Zn-MCSNs demonstrated strong impacts against bacteria in vitro and in vivo, without compromising biocompatibility and hardness of materials.	2021, 2020	[20,186]
Epoxy resin	AH 26 (Dentsply Sirona, Germany)	Chitosan NPs	Antimicrobial activity (*E. faecalis*)	Incorporation of chitosan NPs (10, 20, and 30%) produced a larger inhibition zone with improved cytocompatibility.	2022	[160]
	AH Plus (Dentsply Sirona, Germany)	Bismuth lipophilic (BisBAL) NPs	Antimicrobial activity (*E. faecalis*)	Incorporation of BisBAL NPs produced a larger inhibition zone and inhibited biofilm formation completely on both the *E. faecalis* ATCC strain and the clinical isolates from endodontic patients.	2022	[235]
	AH Plus (Dentsply Sirona, Germany)	Ag NPs	Antimicrobial activity (*Klebsiella* and *E. coli*, and *E. faecalis*)	The incorporated Ag NPs did not prevent apical bacterial leakage after 3 months. Ag NPs-loaded sealers exhibited comparable antibacterial activity to those added with chitosan NPs.	2021, 2019	[114,120]
	AH Plus (Dentsply Sirona, Germany)	Chitosan NPs	Antimicrobial activity (*E. faecalis*)	Chitosan NP-loaded sealers had comparable antibacterial activity to those with Ag NPs.	2019	[114]
	ADSEAL (META BIOMED, Korea)	Chlorhexidine (CHX)/Ag NPs @ multi-walled carbon nanotubes (CNTs)	Antimicrobial activity (*E. faecalis*, *C. albicans* and *S. aureus*)	Addition of CNTs incorporating CHX and Ag NPs enhanced antibacterial and antifungal efficacy.	2021	[180]
	AH Plus (Dentsply Sirona, Germany)	Quaternary ammonium polyethyleneimine NPs	Antimicrobial activity (*E. faecalis*)	The incorporation of quaternary ammonium polyethyleneimine NPs significantly improved antibacterial activity.	2015	[173]
	AH Plus (Dentsply Sirona, Germany)	AgVO_3_ nanomaterials	Biocompatibility (human gingival fibroblast)	A reduction in cell viability was due to AgVO_3_.	2021	[229,230]
	Sealer 26 (containing calcium hydroxide; Dentsply Sirona, Brazil)	AgVO_3_ nanomaterials	Biocompatibility (human gingival fibroblast)	A reduction in cell viability was due to AgVO_3_.	2021	[229,230]
	AH Plus (Dentsply Sirona, Germany)	Mg(OH)_2_ NPs	Biocompatibility (MC3T3-E1 cells), bioactivity and antimicrobial activity (*S. mutans*)	The addition of 3% Mg(OH)_2_ NPs promoted cell proliferation and osteogenic differentiation. 5% and 7% Mg(OH)_2_ NPs enhanced the antibacterial function of AH Plus in the fresh state.	2020	[146,147]
	AH Plus (Dentsply Maillefer, USA)	β-TCP nanocrystals	Biocompatibility (human periodontal ligament fibroblasts), antimicrobial activity (*E. faecalis*, *C. albicans*, *E. coli*) and physiochemical property	The addition of β-TCP did not affect antibacterial activity, but supported higher cell viability as well as increased adhesiveness to root canal walls.	2019	[236]
	AH Plus (Dentsply Sirona, Germany)	Fluoridated bioactive glass NPs (F-nBG)	Physiochemical property	F-nBG incorporated sealers released fluoride and gave enhanced bond strengths.	2020	[142]
	AH 26 (Dentsply Sirona, Germany)	Fluoridated hydroxyapatite NPs, hydroxyapatite NPs and bioactive glass NPs	Physiochemical property and bioactivity	BAG and HA NPs enhanced the in vitro apatite-forming ability and did not alter the physical performance of AH 26. However, fluoridated HA NPs did not improve the apatitic layer formation.	2019	[139]
	AH Plus (Dentsply Sirona, Germany)	ZnO NPs	Penetrability to dentinal tubule	Addition of ZnO NPs significantly enhanced tubular sealer penetration depth.	2020	[131]
Methacrylate resin	Lab-made experimental materials	Ag@SiO NPs	Antimicrobial activity (*E. faecalis*)	The addition of Ag@SiO up to 10 wt % did not affect the biocompatibility, radiopacity, flow, film thickness, and showed an immediate and long-term (9 months) antibacterial effect.	2023	[2]
	Lab-made experimental materials	Calcium hydroxide-containing halloysite nanotube (HNT_CaOH2) and β-tricalcium phosphate-containing nanotube (HNT_β-TCP)	Antimicrobial activity (unknown species)	The incorporation of HNT_CaOH2 or HNT_β-TCP reduced the bacterial count.	2022	[178]
	Lab-made experimental materials	Halloysite nanotubes (HNT) doped with alkyl trimethyl ammonium bromide(ATAB)	Antimicrobial activity (*E. faecalis*)	The incorporation of ATAB/HNT enhanced antibacterial activity against biofilm and planktonic *E. faecalis.*	2019	[177]
	Lab-made experimental materials	ZnO NPs with needle-like nanostructure	Antimicrobial activity (*E. faecalis*)	ZnO NPs improved the antibacterial effect without a significant detrimental impact on the chemical and physical properties.	2020	[124]
	EndoREZ (Ultradent, USA) with 2.5% quaternary ammonium salt	Magnetic nanoparticles or Fe_3_O_4_ NPs	Penetrability to dentinal tubule and antimicrobial activity (*E. faecalis*)	Fe_3_O_4_ NPs could penetrate into dentinal tubes under a magnetic field to kill bacteria embedded in the deeper dentinal tubules in vitro and in vivo.	2022	[132,237]
	Lab-made experimental materials	Hydroxyapatite NPs	Bioactivity	Hydroxyapatite NPs were inferior to α-TCP in terms of stimulating mineralized nodule formation.	2021	[137]
	Lab-made experimental materials	Amorphous calcium phosphate NPs (ACP NPs)	Mineralization	The incorporation of ACP NPs increased the release of Ca and P ions at pH levels below 7.	2019, 2017	[148–150]
Others	Lab-made experimental materials (polyurethane base)	nano-ZnO and nano-hydroxyapatite	Antimicrobial activity (*S. mutans*, *S. aureus*, and *E. faecalis*) and biocompatibility (L929 cells)	Zn-containing sealers exhibited more robust and long-lasting antibacterial activity and lower cytotoxicity than the Ag-containing sealers. Nano-HA exhibited stable antibacterial activity.	2021, 2019	[126,127]
	Lab-made experimental materials (urethane-acrylate base)	Nanoscale silicate platelets (NSPs) immobilized with Ag NPs and/or ZnO NPs (Ag@NSP, ZnO@NSP, or Ag/ZnO@NSP)	Antimicrobial activity (*E. faecalis*) and biocompatibility (3T3 cells)	Simultaneous immobilization of Ag NPs and ZnO NPs on silicate platelets enhanced the antibacterial activities, and also reduced the dose of Ag NPs needed, resulting in acceptable cytotoxicity.	2020	[125]
	Lab-made experimental materials*	Monodispersed silica-based bioactive glass NPs (SBG-NS) grafted with quaternary ammonium polymethacrylate (QAPM)	Antimicrobial activity (*E. faecalis, S. mutans*, *and S. sanguis*) and biocompatibility (periodontal ligament stem cells, calvarial implantation model)	SBG-QAPM had the strongest long-term antibacterial effect and lowest inflammatory reaction when compared with ProRoot MTA, Endomethasone C, and AH Plus.	2017	[151]
	Lab-made experimental materials*	Polymeric PLGA NPs loaded with Egyptian propolis extract (ProE)	Biocompatibility (subcutaneous implantation model) and sealing ability	ProE-loaded PLGA NPs caused milder inflammatory reactions and gave comparable sealing ability when compared with AH Plus.	2020	[188]

*The main compositions of sealers are listed nanomaterials.

### Metal/metal oxide NPs as endodontic sealers

The rationale for including Ag NPs into endodontic sealers is that they exert antifungal actions against *C. albicans* and antibacterial actions against many bacterial species, including *S. aureus*, *S. mutans*, *Streptococcus pyogenes*, *P. vulgaris*, *E. coli*, and *E. faecalis* [111–113]. The approach of adding Ag NPs (54.2 nm) into endodontic sealers to enhance their antibacterial activity against *E. faecalis* has been effective for multiple sealers (AH Plus, Endosequence, MTA Fillapex, Sealapex and Tubliseal) [114]. Also, both Ag NPs (20 nm) and other antimicrobial agents such as dimethylaminohexadecyl methacrylate (DMAHDM) have been incorporated into a commercial sealer (AH Plus) [115,116]. A silicone-based (polydimethylsiloxane) sealer has been modified with nanosilver (NanoSeal-S). This gives apical sealing comparable to AH Plus, but its bond strength and sealing ability are inferior to BioRoot RCS [117–119]. Nevertheless, even sealers with Ag NPs will not block bacterial leakage over long timeframes such as 3 months [120].

Since sealers may potentially come into contact with periapical tissues, issues of irritation and toxicity must be considered, e.g., Ag NPs could trigger the generation of ROS and thus cause nonspecific oxidative damage [121]. Silver ions that are released could also influence bone-derived cells, as shown in cell culture experiments with Ag NPs using osteoblastic MC3TC E1 cells, in a dose-dependent manner [20], and likewise for AgVO_3_ NPs when tested using human gingival fibroblasts [122].

Zinc oxide NPs exert antibacterial actions by disrupting bacterial cell membranes [14]. Compared to its bulk state, ZnO at nanoscale displays superior bactericidal effects, because of the large surface area of the NPs [122]. ZnO NPs exhibited stronger antimicrobial actions than NPs of other metal oxides (e.g., MgO, TiO_2_, Al_2_O_3_, CuO, and CeO_2_) [123]. ZnO NPs (~40 nm) have been incorporated into methacrylate resin-based sealers, and this inhibits the growth of planktonic *E. faecalis* without compromising the physical performance of the original sealers [124]. Interestingly, materials with ZnO NPs (~30 nm) appear to be less cytotoxic to fibroblasts than their Ag NPs-containing counterparts [125–127]. Adding Ag NPs may reduce the viability of MC3T3 E1 cells in culture after 3 days, while in contrast adding ZnO NPs maintains or slightly promotes cell proliferation in culture. If both types of NPs are used together, it may be possible to optimize the antibacterial effects and cytocompatibility by adjusting the ratio between Ag NPs and ZnO NPs [20]. However, ZnO NPs (<50 nm) could be toxic for osteoblasts, whereas no greater than ZnO microparticles [128]. In general, cytotoxicity varies based on NP shape and size, and the type of cell that is being exposed to the NPs [129]. Therefore, it is logical to consider host cells that reside in the periapical area, including periodontal ligament cells, alveolar bone cells, and immune cells when designing endodontic products.

Adding NPs to sealers may also enhance their physical properties. One study showed that replacing ZnO powder with ZnO NPs (~20 nm) significantly improved the physicochemical properties of conventional Grossman sealer in terms of flow, setting time, dimensional stability, and radiopacity [130]. Likewise, adding ZnO NPs into AH Plus enhanced the flow characteristics and favors deeper penetration into dentinal tubules [131]. Other metal or metal oxide NPs are also of interest as materials that could be added into existing root canal sealers. One recent study incorporated ferrimagnetic magnetite (Fe_3_O_4_) NPs (50 to 100 nm) and a quaternary ammonium salt (QAS) into EndoREZ sealer [132]. By applying an external magnetic field, dentinal tubule penetration was enhanced.

### Bioceramic nanomaterials as endodontic sealers

Ceramics are inorganic nonmetallic solids that exist in amorphous or crystalline forms. Their elemental composition includes primarily calcium, silica, phosphorous, and aluminum [133]. ​A consideration with endodontic sealers is that when extruded they will come into contact with periradicular tissues. Many commercially available sealers are based on epoxy resins or methacrylate resins and do not have the ability to drive hard tissue formation because they lack bioactive components. More recently, research into nanophase bioceramics has been carried out to identify their prospects in optimization of endodontic sealers [134]. It was reported that the addition of nano-hydroxyapatite (nHA) (~26.8 nm) in methacrylate-based root canal sealers, even up to 40%, had no negative effects on radiopacity, flow, and film thickness [135]. Furthermore, there is a consensus among the literatures that nHA could enhance in vitro apatite-forming ability of pristine endodontic sealers [136–138]. Apart from nHA, nanostructured bioactive glass or bioglass (nBAG), an amorphous silicate-based compound, also has exhibited excellent capability to induce apatite formation and osteogenic differentiation [139–141]; moreover, the sealing ability of certain endodontic sealer could be promoted by fluoridated nBAG [142]. However, previous data suggested the inferiority of weak antibacterial effect of nHA and nBAG [140,143,144]. It would thus be of clinical interest to converge desired properties for endodontic sealers, i.e., bioactivity and microbicidal effect, in cases of severe pulpal and periapical inflammation [145]. Recently, nano-magnesium hydroxide containing AH Plus sealers were shown to exhibit improved bioactivity and antibacterial effects against *S. mutans* [146,147]. Beside incorporating nanobioceramic alone, some researchers also attempted to introduce effective antibacterial component 12-methacryloyloxydodecylpyridinium bromide (MDPB) and/or DMAHDM together with amorphous calcium phosphate (ACP) NPs to develop novel bioactive endodontic sealers with mineralization and antibiofilm properties [148–150]. The results demonstrated bioactivity of modified sealers to release abundant Ca and P ion, and the antibacterial activity to inhibit *E. faecalis* biofilm as well as multi-species biofilm. Similarly, new strategy to permanently copolymerize antimicrobial quaternary ammonium methacrylate salt (QAMS) with endodontic sealers based on monodispersed silica-based bioactive glass (SBG) nanospheres has also been reported [151]. SBG sealers covalently grafted with QAMS demonstrated excellent stability after 6-month immersion in phosphate-buffered saline (PBS) and a level of biocompatibility that was comparable to ProRoot MTA, and superior to Endomethasone C and AH Plus.

With bioceramic sealers turning into the focal point, more efforts were made to provide an added value for them, by adequate modification [134]. To address the limitation of slow hydration nature of dicalcium silicate-based sealers, Zheng *et al.* [152] explored the influence of attapulgite (ATT) nanofiber, a clay that belongs to hydrous Mg–Al–silicate minerals, on the self-curing of prime cement sealers. Data obtained showed that ATT nanofiber with low content (≤4%) not only contributed to structural densification and favored self-curing property but also enhanced compressive strength and improved anti-microleakage ability. Baghdadi *et al.* [153] in their study evaluated the reinforcement of 1 and 2 wt % multi-walled carbon nanotubes (MWCNTs), titanium carbide (TiC), and boron nitride (BN) separately in BioRoot RCS, a typical calcium silicate-based root canal sealer. The resultant sealer demonstrated a shorter setting time, lower elution profiles, and high alkalinity with a pH ≥10 after 24 h, which are beneficial for minimizing microleakage and inhibiting microbes. Further scanning electron microscopy (SEM) analysis showed that nanomaterials with the lower percentage (1%) were more homogeneously distributed when compared to the ones with higher concentration (2%). Besides, different radiopacifiers at nanoscale such as zirconium oxide (ZrO_2_) and niobium oxide (Nb_2_O_5_) were also utilized to improve the overall physicochemical properties and bioactivity of root canal sealers [154,155].

### Organic NPs as endodontic sealers

Organic NPs such as chitosan NPs have attracted considerable attention in endodontic therapy because of their notable biocompatibility and broad-spectrum antimicrobial activities [156]. Around the early 2010s, their antibacterial properties and in vitro studies to simulate clinical scenario for endodontic sealer application began to emerge linking [157]. It was reported that incorporating chitosan NPs into epoxy-based sealers could significantly enhance the antibacterial properties, as a result of both direct-contact and membrane-restricted antibacterial assays [158]. Furthermore, the addition of chitosan NPs prolonged the antibacterial efficacy of commercial endodontic sealers even after a 1-month duration by reducing total and viable biovolume of *E. faecalis*. To evaluate the significance of chitosan NPs in terms of antimicrobial effectiveness, researchers incorporated chitosan NPs alone or together with CHX in various conventional endodontic sealers, and further compared them with those containing Ag NPs or CHX [114]. Data from bacteriostasis circle and SEM micrographs have identified that the synergistic bactericidal activity of chitosan NPs and CHX had the strongest inhibition against *E. faecalis*, irrespective of the types of sealers. However, one study by Nair *et al.* [159] showed that the incorporation of chitosan NPs in Ca(OH)_2_ sealer (i.e., Apexit Plus) failed to improve its antibacterial properties against *E. faecalis* strain OG1RF. Moreover, Ratih *et al.* [160] found that the increment of chitosan NP concentration from 10% to 30% in epoxy resin-based sealer (AH-26) did not significantly enlarge *E. faecalis* inhibition zone, indicating the need for more comprehensive investigations to optimize the antimicrobial effects of chitosan NPs.

Achieving an ideal endodontic sealer involves addressing the challenge of preventing collagen degradation, which can compromise interfacial adhesion, promote microbial recolonization, and reduce instrumented dentin resistance to fracture. Persadmehr *et al.* [93] demonstrated that collagen gels treated with 1% chitosan NPs exhibited reduced hydroxyproline release in the presence of collagenase, suggesting chitosan NPs’ potential to protect collagen from enzymatic degradation. Further SEM analysis indicated the intimate association of chitosan NPs with collagen fibrils [161]. Recent evidence highlights the ability of chitosan NPs-containing nanocomplexes, such as carboxymethyl chitosan/ACP nanocomplexes (<40 nm), to achieve intrafibrillar mineralization of collagen, enabling dentine repair. Employing this biomimetic approach, chitosan/HA nanocomposites blended with tricalcium silicate sealer improved the physico-mechanical properties of endodontic sealers and enhanced root dentin fracture resistance [162,163].

Furthermore, quaternary ammonium compound (QAC) NPs [164,165], such as quaternary ammonium polyethylenimine (QPEI) NPs [166], exhibit robust antibacterial properties. QPEI NPs, immobilized on resinous material surfaces, demonstrate enduring antimicrobial function without leaching [167]. Studies by Beyth *et al.* [168] reveal that QPEI NPs enhance antibacterial activity against *E. faecalis* without compromising physical properties or setting time. QPEI NP modification in commercial endodontic sealers, including AH Plus, GuttaFlow, and Pulp Canal Sealer EWT, also improves antibacterial effects [169–171]. In addition, Shvero *et al.* [167] introduced an antigravitational test using endodontic sealers and a bacterial pellet to investigate the cellular behavior of anionic *E. faecalis* on cationic QPEI NPs-modified sealer surfaces. The study revealed that QPEI NPs attracted *E. faecalis* through electrostatic interactions, leading to dead cells observed throughout all layers of the formed biofilm, suggesting both direct and indirect mechanisms for QPEI NPs in bacterial eradication.

To better understand latent toxicity of QPEI NPs on periapical tissues, Barros *et al.* [172] in their study evaluated the proliferation, apoptosis, and phenotype differentiation of human osteoblastic and osteoclastic cells, which had been exposed to extracts from pristine and QPEI NP-containing AH Plus and Pulp Canal Sealer EWT. Additionally, confocal laser scanning microscopy (CLSM) results from evaluating AH Plus sealers containing 1% QPEI NPs in an infectious root canal model demonstrated successful bacterial infiltration in dentinal tubules and reduced bacterial viability upon root canal obturation with 1% QPEI NPs-containing endodontic sealers [173]. Furthermore, to increase the affinity of QACs with epoxy resin-based AH Plus, Gong *et al.* [174] synthesized quaternary ammonium epoxy silicate (QAES) NPs, which enables copolymerization of these NPs with epoxy resin-based materials. Direct-contact test and CLSM observations indicated that even after 4 weeks of water aging, QAES NPs-incorporated endodontic sealers possessed antibacterial activity against *E. faecalis* in the form of both plankton and biofilm, suggesting their superiority over unmodified controls in the long term.

Moreover, QACs, including QPEI NPs, exhibit the ability to inhibit matrix metalloproteinases (MMPs) impact on dentin collagen when incorporated into dental materials as polymerizable quaternary ammonium methacrylates, offering a sustained anti-MMPs effect [175]. Molecular interrelationships between QACs and MMPs catalytic sites hinder collagen matrix hydrolysis [164]. Considering the crucial role of MMPs in controlling infections, QAC NPs may be advantageous for reducing periapical inflammation. Comprehensive studies are needed to further understand the value of QAC NPs-containing endodontic sealers in inhibiting bacteria, sealing root canals, and promoting periapical inflammation healing.

### Nanomaterials as nanocarriers for sealers

Nanomaterials, such as halloysite nanotubes (HNTs) and MWCNTs, serve as promising nanocarriers for endodontic sealers. HNTs, with their unique tubular structure [176], successfully encapsulate antimicrobial compounds, enhancing the antibacterial efficacy of methacrylate resin-based root canal sealers [177]. Additionally, the incorporation of calcium-loaded HNTs demonstrates a significant reduction of *E. faecalis* when compared with AH Plus and pristine experimental sealers, highlighting the potential of clay nanotubes in endodontic applications [178]. Meanwhile, MWCNTs, explored for physical reinforcement in BioRoot RCS [179] and as carriers for CHX and Ag NPs in ADSEAL [180,181], exhibit uniform distribution and promising long-term antimicrobial effects. Specifically, even after 7-day setting, CHX and Ag NPs-encapsulated MWCNTs exhibited satisfactory antimicrobial effects, with the maximum efficacy against *E. faecalis*, followed by *S. aureus* and *C. albicans*. However, the impact of different CNTs on endodontic bacterial inactivation and sealer physical properties requires further investigation.

Mesoporous silica nanoparticles (MSNs) are extensively studied as nanocargoes due to their remarkable biocompatibility, large drug-loading capacity, and customizable physicochemical properties [182]. Li *et al.* [183] demonstrated that MSNs with a diameter below 100 nm effectively infiltrate dentin tubules, showcasing potential for delivering antimicrobial and mineralization agents in endodontic sealers. Similarly, mesoporous calcium-silicate NPs (MCS NPs) exhibit promise for infiltrating dentinal tubules [184]. Continuous release of Ca^2+^ and SiO_4_^4−^ creates a microenvironment inhibiting microbes [20,185]. In vivo root canal infection model further illustrated that MCS NPs loaded with nano-silver and nano-zinc could effectively reduce inflammatory area in the periapical tissue, suggesting their prospects to be utilized in endodontic sealers [186]. Rucker *et al.* [2] extended the antimicrobial effect of endodontic sealers by incorporating Ag@SiO_2_ NPs, confirming reduced *E. faecalis* viability while maintaining sealer physicochemical properties. More recently, nanoscale silicate platelets (NSPs) immobilized with Ag NPs and/or ZnO NPs were prepared to render homogeneous dispersion of NPs while maintaining the microbicidal efficacy of Ag NPs and ZnO NPs in resin-based endodontic sealers [125].

Polymer-based NPs, such as polylactic-co-glycolic acid (PLGA) NPs, enhance pharmaceutical delivery and release. Pagonis *et al.* [187] found PLGA NPs concentrated on *E. faecalis* cell walls, developing a nanoplatform for PDT in infected root canals. Raheem *et al.* [188] introduced propolis-loaded PLGA NPs with prolonged propolis release, exhibiting satisfactory cytocompatibility and robust antimicrobial activity against *E. faecalis*, *S. mutans*, and *C. albicans*.

## Nanomaterials Used as Retro-Filling and Root-Repair Materials

Several studies have emphasized the significance of placing root-end fillings during periapical surgery [189,190]. The material chosen for root-end filling significantly impacts procedural success, aiming to seal the root end effectively [191]. Mineral trioxide aggregate (MTA) remains the most widely used and is still considered the gold standard for comparison with new materials [192]. However, MTA has drawbacks, including handling difficulties, a prolonged setting time, high cost, a lack of antimicrobial properties, and susceptibility to dissolution in acidic pH [193]. Incorporating NPs into MTA and other bioactive endodontic cements offers the potential to enhance their mechanical, physical, and biological properties.

Incorporating Ag NPs into bioactive endodontic cements yields multifaceted improvements. First, it substantially enhances antimicrobial activity in materials like MTA and calcium-enriched mixture, effectively combating bacteria linked to dental infections without compromising the cements’ physical properties [194]. Furthermore, it demonstrates excellent tissue reaction and biocompatibility, as observed in a rat model, with no significant adverse effects in subcutaneous tissue compared to control implants without Ag NPs [195]. Additionally, Ag NPs addition increases calcium ion release from MTA, crucial for promoting new bone tissue growth and expediting the healing process [196].

Moreover, incorporating Ag NPs is reported to reduce the setting time of MTA and accelerate silicates’ hydration and potentially enhance the material’s utility [197]. It is also reported to improve dimensional stability, vital for proper tissue regeneration, and serves as an excellent radiopacifier, overcoming MTA’s low radiopacity for clearer radiographic observations [196,198].

Various other NPs have also been investigated for their potential to enhance endodontic cements. The introduction of bismuth lipophilic NPs into MTA has shown a significant improvement in antimicrobial and antibiofilm activities without compromising physical properties, promising enhanced clinical outcomes [199]. Silver-zeolite NPs, known for their porous structure releasing silver ions over time, augment the antibacterial properties of MTA against diverse microorganisms. However, potential drawbacks such as altered solubility and pH changes necessitate careful consideration for clinical applications [200–204]. The incorporation of TiO_2_ NPs into MTA at 1 wt % demonstrates favorable biocompatibility without adverse effects. This addition improves flexural and compressive strength, showcasing potential material property enhancements [205–207]. ZnO NPs combined with Portland cement (PC), alongside zirconium oxide, exhibit antibiofilm activity and radiopacity. However, a cautious approach is essential, as higher concentrations of ZnO NPs may compromise compressive strength, demanding a thoughtful material selection process in various clinical scenarios [208]. Incorporating hydroxyapatite NPs negatively affects compressive strength and solubility but enhances radiopacity, final setting time, and antibiofilm activity. These trade-offs require careful consideration, particularly regarding water requirements and clinical acceptability [209].

Bond strength is a critical determinant in endodontic repair materials. Notably, the incorporation of TiO_2_ and Ag NPs into MTA has been found to enhance push-out bond strength, whereas the introduction of silicon dioxide NPs demonstrates no significant impact [210]. A comparable investigation reveals that the bond strength of calcium enriched mixture (CEM) cement remains unaffected by the addition of TiO_2_, whereas the inclusion of TiO_2_ in MTA and PC leads to an increase in push-out bond strength for these materials [211].

## Nanomaterials Used for Pulpal Repair and Regeneration

Pulpal regeneration procedures, which aim to biologically and functionally repair the pulp–dentin complex, require appropriate disinfection of the root canal space, followed by pulp tissue repair [212]. Regenerative medicine is based upon three key elements: stem cells, natural/synthetic scaffolds, and growth factors (bioactive molecules). Stem cells from different sources have been studied for the purpose of regenerating pulp and dentin. These sources include dental pulp stem cells, stem cells from the apical papilla, stem cells of human exfoliated teeth, and periodontal ligament stem cells [213]. Scaffolds, resembling the structure and complexity of the extracellular matrix in the pulp, offer mechanical support, facilitate cell attachment, and stimulate the growth and differentiation of stem cells. Bioactive molecules are signaling compounds that play a crucial role in initiating and sustaining cellular interactions necessary for the regeneration of damaged pulp tissues. To facilitate tissue regeneration, these molecules rely on a suitable microenvironment and an appropriate scaffold [214].

### Nanofibrous-based scaffolds for pulpal repair and regeneration

In order to regenerate pulp and dentin successfully, scaffolds should mimic the extracellular matrix, extracellular fibers, and proteins of pulp and dentin; collagen, which constitutes the majority of dentin, is an excellent natural scaffold [215]. Nanofibrous scaffolds have been found to promote positive interactions between cells and the extracellular matrix, enhance cell proliferation, maintain cell phenotype, facilitate stem cell differentiation, and activate cell-signaling pathways through physical and chemical stimuli [216,217].

Several mechanisms have been proposed to explain the positive biological effects induced by nanofibers. These mechanisms include increased protein adsorption, enhanced integrin expression, and altered signaling pathways. It is suggested that nanofibers promote initial cell attachment by selectively absorbing extracellular matrix proteins such as fibronectin, vitronectin, and laminin. This increased protein adsorption may subsequently lead to higher expression of integrins, which are proteins involved in cell–extracellular matrix attachment [217]. Studies have shown that nanofibrous matrices can up-regulate the expression of specific integrins in cells, which has been linked to enhanced differentiation into mesodermal and osteogenic lineages. Integrins may activate proteins like paxillin and focal adhesion kinase, which play roles in differentiation pathways. Additionally, the expression of RhoA and Rac, which regulate cytoskeletal organization and other cellular behaviors, has been associated with differentiation, morphology, and adhesion responses [217].

### NP-based systems for pulpal repair and regeneration

Nanotechnology offers a promising avenue for developing scaffolds composed of fibers containing bioactive NPs in the context of regenerative endodontics [218]. These NPs possess unique attributes, such as small size, high surface area, increased solubility, and antibacterial activity, making them valuable for controlled release of bioactive agents [219]. Notably, magnetic NPs, like superparamagnetic iron oxide NPs and quantum dots, serve for long-term tracking of stem cells after transplantation, providing insights into their migration and differentiation in dental pathology and repair [220]. Incorporating iron oxide NPs into a calcium phosphate cement scaffold demonstrated enhanced attachment of human dental stem cells and facilitated bone mineralization [220]. Similarly, titanium dioxide NPs proved beneficial by improving the proliferation and morphology of dental pulp stem cells without causing cytotoxicity, contributing to collagen fiber formation essential for hard tissue development [221].

Hydroxyapatite NPs exhibited positive effects on stem cells from the apical papilla, enhancing adhesion, proliferation, and odontoblast differentiation on scaffolds [222]. Mesoporous bioactive NPs stimulated odontoblast differentiation from rat dental stem cells, showcasing potential applications in dental materials for damaged dentin regeneration [223]. Zinc bioglass NPs in calcium phosphate cement promoted odontoblast differentiation and angiogenesis, suggesting a role in regenerative dentistry [224]. Chitosan NPs, recognized for drug delivery, demonstrated varying impacts on stem cells from the apical papilla, influencing alkaline phosphatase activity and odontogenic differentiation based on the encapsulation or adsorption techniques [225,226].

Furthermore, mesoporous bioglass nanospheres, with the ability to deliver silver ions, antimicrobial drugs, and regenerative ions, present a multifaceted approach for antibacterial and pro-regenerative effects. This NP system, featuring intracellular delivery, ionic doping capabilities, high mesoporosity, degradability, and biocompatibility, holds promise for repairing and regenerating infected tissues [227].

## Conclusion and Prospects

In conclusion, the growing body of research exploring nanomaterials in endodontics underscores their potential as valuable tools in combatting root canal infections and promoting endodontic tissue repair. The antibacterial, anti-adhesive, and drug delivery capabilities of NPs present promising avenues for enhancing endodontic treatments. However, it is crucial to acknowledge that the current evidence is primarily based on preliminary reports, warranting additional investigations through animal and in vivo studies. To establish the clinical validity of these findings and ensure the safety of nanomaterial applications, further research efforts should focus on elucidating cytotoxicity profiles and thoroughly assessing the biological effects of these innovative approaches. This ongoing exploration holds the key to realizing the full potential of nanomaterials in advancing the field of endodontics and improving patient outcomes.

Understanding the potential drawbacks and long-term toxicity of nanomaterials is paramount given their diverse pharmacodynamic effects. The escalating utilization of NPs raises concerns about their environmental accumulation and unintended impacts on ecosystems. Cellular repercussions, including apoptosis induction, inhibition of cell growth, genotoxicity, and neurological and respiratory damage, necessitate careful consideration in NP applications. Nanosized particles exhibit heightened inflammatory, oxidative, and cytotoxic effects compared to larger counterparts, owing to increased lung deposition and systemic translocation [8]. Technologically, stability, biodistribution, degradation, and interactions with body fluids and cells are critical considerations. Consequently, strict quality control procedures, comprehensive risk assessments, and utmost vigilance are imperative in the realm of nanotechnology research [228].

Furthermore, the majority of research on nanomaterials’ impact on oral pathogenic biofilms has been conducted in vitro, predominantly focusing on common oral bacteria within single-species biofilm models. This approach overlooks the complexity of biofilms composed of multiple species, including fungi and viruses, which are also present in oral biofilms. Notably, investigations into the effects of NPs on oral pathogenic biofilms have primarily concentrated on bacteria, potentially yielding conclusions that differ from real clinical scenarios. Addressing these limitations is crucial for a more comprehensive understanding of the implications of nanomaterials in the context of oral health [67].

In addition, a substantial challenge in the clinical application of nanomaterials in endodontics lies in the necessity for comprehensive large animal and clinical trials. While initial investigations show promising outcomes in vitro and in smaller-scale studies, the translation of these findings to real-world clinical scenarios demands rigorous evaluation through extensive trials involving larger animal models and human subjects. Large animal trials provide a crucial intermediary step, offering insights into the biological responses, safety profiles, and potential efficacy of nanomaterials in a more complex biological environment. Subsequently, clinical trials are indispensable for validating the feasibility, safety, and efficacy of these nanomaterials in diverse patient populations, ensuring that the proposed innovations meet the stringent standards required for successful integration into routine endodontic practices. The transition from laboratory findings to clinical reality necessitates a meticulous and stepwise approach, emphasizing the importance of robust evidence derived from large-scale trials for the successful implementation of nanomaterials in endodontic care.
